# A Treatise on the Nature and Cure of Gout and Rheumatism, Including General Considerations on Morbid States of the Digestive Organs; Some Remarks on Regimen; and Practical Observations on Gravel

**Published:** 1820-01-01

**Authors:** 


*A Treatise on the Nature and Cure of Gout and Rheumatism, including General Considerations on Morbid States of the Digestive Organs; some Remarks on Regimen; and Practical Observations on Gravel.* By CHARLES SCUDAMORE, M. D. Member of the Royal College of Physicians, &c. London. One Volume Octavo, 734 Pages. Third Edition, revised and materially enlarged. London, 1819.

AMONG the very few *medical* authors who, like the Bard of the North or the Exile of the South, have realized the fable of Midas, and converted paper into gold by the magic touch of their pens, Dr. Scudamore may, we think, be numbered. We do not speak of the direct transmutation, as it would be understood in Pater-Noster Row by the adepts in the trade. Far from it! Small indeed is the return of "shining ore," from those great granaries of literature, even although second and third editions roll majestically into them—*facilis descensus averni!*—many an author, reputedly successful, could add with strict *truth*,—

Non unquam gravis ære domum mihi dextra redibat!

But it is the indirect process of *aurifying* paper through the medium of that powerful engine—the press, which is the grand secret. Notwithstanding the occasional exceptions to this, as to all other general rules, nothing can be more true, than that, in the medical profession, as elsewhere, "knowledge is power," and a good name the straightest road to riches. It need hardly be stated, for it is almost universally admitted, that the PRESS is the most effectual and certain channel for the direction of real knowledge and solid talent. Merit cannot there be jostled out of the path to reputation, by impostors and pretenders, who seek only for emolument. The latter class, indeed, are so well aware of this truth, that, daring not to expose their real character by a public exhibition or competition, they take all possible means of depreciating and stigmatizing talent and literary acquirements, under the names of genius, book-learning, theories, &c. Their efforts, however, are generally ineffectual; for such is now the diffusion of knowledge, that merit becomes quickly recognized, through the medium of the press, and that to an extent which a whole life of labour would not otherwise reach. "Quo mihi rectius videtur, ingenii, quam virium opibus gloriam quærere; et quoniam vita ipsa, quâ fruimur, brevis est, memoriam nostri quam maxime longam efficere. Nam divitiarum et formæ gloria, fluxa atque fragilis est; virtus clara æternaque habetur."

*Sallustius.*

But the press, like the Chariot of the Sun, is a most difficult vehicle to manage, and many a hapless wight experiences therefrom, the fate of the ambitious Phæton. A man may proudly soar on Icarian wings, but the light of public discussion and feeling soon dissolves the wax, and a precipitation follows.* In committing ourselves to print, we spread a table, where every guest may seat himself, whether friend or foe, and ask questions, or make comments, without the slightest control. The task of authorship, therefore, should not be rashly undertaken. We should weigh well the consequences, before we embark on this sea of troubles; for having once embarked, we have no longer a friendly port into which we can steer for shelter and obscurity; we must weather the storm by means of our own resources, or sink in the conflict. "Nam, et priusquam incipias, consulto, et ubi consulueris, maturè facto opus est." *Sall.*

---

* We have been accused of using language too figurative. But let it be remembered, that the *Review* of a work involves occasionally reflections, allusions, and digressions that are, in their nature, very different from what may be termed purely medical matter, and consequently require a different language and style to express them with force and effect. Whenever the subject matter becomes grave and philosophical, the language, of course, should correspond, and derobe itself of all other ornament than correctness and perspicuity. We conceive, however, by the way, that the study of medical literature has suffered some retardation by the barbarous, uncouth, and slovenly language in which it has been too often the custom for medical authors to write. How disgusting this language appears to a youth who has just emerged from the study of Grecian and Roman lore, those who have any knowledge of classic elocution can say. We have seen numerous instances of that disgust continuing to operate till a period of life, when it was too late to retrieve the misfortune. But to return to the subject of this Note. We would be glad to know, by what rule we are bound to adhere to the same precise and philosophical language, when describing the influence of the press on the progress of science, which we would use in describing the influence of a nerve on the motion of a muscle?

It may be objected, that many works are published which escape without either censure or applause. But in this case, silence is the deadliest satire, since to excite animadversion itself is, in these times, some proof of merit.

We were going to say, that Dr. Scudamore was a fortunate author; but fortune or chance has not much to do in medical writings. *Labor, improbus labor,* is the grand engine; for although talent, like gout, is often hereditary, it is often also acquired, and the great apparent inequalities of ability in men, are in three cases out of four, dependent on unequal application. Animus hominis quicquid sibi imperat, obtinet. *P. Syr.* Dr. Scudamore, however, has been fortunate in the choice of his subject. Gout is a rich subject; it is the offspring of wealth in the patient, and the parent of affluence in the practitioner; rivetting the one to his couch, and rolling the other in his chariot. No wonder then that so many volumes have been dedicated to this noble—nay, royal patron.

Dr. Scudamore is, we believe, the *greatest* writer on the disease under consideration, that has appeared in this country. We do not mean any equivoque, in respect to the *size* of the book, but in regard to the labour and research employed in its construction. We cannot, however, but think that he has overdone the business in this third edition. The "obscurus fio" sometimes results from the "brevis esse laboro"; but it much more frequently follows diffuse, laboured, and ultra-minute description. Besides, Dr. S. has now swelled the work to a price and size, that inevitably confine its circulation to the trunks, and exclude it from the ramifications of the profession. This is a great misfortune, and it is all owing to our author's over anxiety to leave nothing unsaid or undescribed on the subject. A single passage from Dr. Scudamore's symptomatology will elucidate this remark.

Thus some pages are taken up in describing the *kinds* of pain, and the metaphorical expressions which patients use under the tortures of gout.

> "Observation has taught me, that the sense of *weight* and total loss of power, are most severely felt, when the whole of the *anterior part of the foot* is the seat of disease; that inflammation in the *first* joint of the great toe produces the *strongest throbbing;* and that the sense of *tightness* is most urgent when the *elbow* joint, and the tendons at the wrist are the parts affected." 32.

Now we would ask Dr. Scudamore the *cui bono* of the information, that *stronger* throbbing attends inflammation of the *first* than of the *second* joint of the great toe? This is brought forward as a fair specimen of the *ramuscular* minuteness with which our author discusses each point of his subject; and probably, like some celebrated *ramusculi* of this capital, he glories in this excessive, but, we would say, unnecessary minuteness.

"Cum gloriaretur quidam quòd multa didicisset; dixit ei Aristippus:—Sicut qui plurima comedunt, non melius valent, quàm qui sumunt necessaria; ita eruditi habendi sunt, non qui *plurima* didicerunt, sed qui *utilia*."—*Diog. Laert.*

In short, we hesitate not to say, and Dr. Scudamore will, we think, ultimately acknowledge the truth of the assertion, that the work under review, like the Roman Empire of old, has lost in strength what it has gained in expansion, and that were he, in a future edition, (and many editions will be demanded) to reduce its dimensions one half, he would increase its utility tenfold.

"Plus prodest, si *pauca* precepta scientiæ teneas, sed illa tibi in promptu et in usu sint, quam si *multa* quidem didiceris, sed illa non habeas ad manum."—*Seneca.*

In the following Analytical Review, it will be necessary to pass over much of what may be termed elementary matter; which, though proper in a professed Treatise on Gout, would be totally misplaced in a Journal of Medicine.

In the arrangement, we think that Dr. Scudamore has greatly improved upon Cullen; and in the history and symptomatology of the disease we have no fault to find, as we before hinted, but that of *prolixity*.

In respect to the Etiology of Gout, Dr. S. has somewhat altered his sentiments of late, on the hereditary nature of the disease. He now attaches more importance to hereditary predisposition than formerly. The beautiful passage in Sydenham is well known, where he reflects that gout "destroys more rich than poor persons, more wise men than fools; which seems to demonstrate the justice and strict impartiality of Providence, who abundantly supplies those that want some of the conveniences of life with other advantages, and tempers its profusion to others with equal mixture of evil."

In the following commentary of Dr. Scudamore on this passage, we cordially agree with its enlightened author.

"Since the period at which these sentiments were entertained, luxury has so much increased among the whole community, that the gout sometimes finds entrance even into humble dwellings. In London, amongst the inferior classes, I have observed butchers, innkeepers, butlers, and porters in wealthy families, to be very subject to gout. It is also frequent among coachmen, chiefly such as live in families; for, together with much excitement from liquors, they are constantly exposed to the variations of the weather. In short, such stations and occupations as most induce repletion and inactivity, or full living, with only passive exercise, lead to gout; and even in some constitutions, in which there is great tendency to plethora and corpulency, moderate indulgence in diet, notwithstanding active exercise, seems to implant the disposition to the disease." 67.

On the predispositions resulting from mental disquietudes, severe study, animal food, strong liquors, indolence, plethora, nimia Venus, and climatorial influence, Dr. Scudamore's remarks are just, but without any thing particularly novel. The section on "a morbid state of the digestive organs, considered both in its particular relation to gout, and in a general view," contains a great deal of excellent matter, and on this section we shall dwell for a few minutes. Dr. S. truly observes, that "the over-excitement of the stomach and chylopoietic functions must lead to relative derangement and debility;" and it is in this, he thinks, that the *acquired*, in contra-distinction to *hereditary* predisposition, principally consists.

"When the stomach is the most affected part, the marks of indigestion are felt in the most sensible manner, by some or all of the following symptoms:—Heartburn; eructations which are sour, attended with a sense of heat, and often conveying the odour of yesterday's meal; a craving appetite, which does not become comfortably satisfied; oppression after a meal, with a painful sense of distention, and soreness of the whole epigastric region. This sense of soreness is sometimes experienced in so extreme a degree, that only slight pressure from dress can be allowed; and even a gentle touch at the ensiform cartilage, or just below that part, is dreaded by the patient as an act of violence. To this account may be added, a furred tongue and clammy state of mouth, with viscid saliva, which is experienced, especially in the first of the morning. Its taste is often remarkably saltish. In dyspeptic persons, in whom the nervous temperament predominates, the tongue is coated either with a white, or yellowish white fur; but in those of the sanguineous temperament, the colour of the fur is a deep brown, or brown mixed with white. In the former class of patients, the complexion of the tongue is very commonly pale; in the latter, quite red. But of all the appearances of the tongue, which indicate a debilitated state of the stomach in its greatest degree, is the cherry-red colour of the whole surface, with more or less of cracks in its substance, and a prominence of the papillæ. By these last characters, the pale tongue also, after a long duration of dyspepsia, is sometimes distinguished, and is to be considered as giving a very unfavourable evidence of the weak powers of the stomach. An extremely clean tongue, with streaks of white and red, is another distinct appearance, as the index of debility. Accordingly, as nervous irritation prevails in the general state of the constitution, a frothy appearance of the tongue and fauces may, in addition to what I have above described, be noticed. Nausea, occasional sickness, flushings after eating a stimulating meal, a giddiness on sudden change of posture, and an uneasy or painful state of head, also occur. With this dyspeptic condition of stomach, the bowels are irregular, but for the most part torpid. Many patients relate that the only predisposing circumstance as introductory to the fit, to which they can make reference, has been a costive state of bowels. The secretion of the urine is variable, both in its quantity and in its properties. Sometimes it is deficient in quantity, of a deep colour, and of high specific gravity; at others, abundant, pale, and much diluted; in which case it is passed with much nervousness and irritation, but without difficulty." 81.

When the chief seat of the complaint is below the stomach, the symptoms are such as are denominated *bilious*, with which a general sallowness, or partial stains of yellowness in the skin, and a dark colour around the lower eyelid, are more or less associated. The appetite, in this case, generally outstrips the digestion. All the secretions become vitiated; the bowels irritable; the peristaltic action irregular; the fæces unnatural in colour and smell.

"Sometimes they are passed as *pellets;* and when more formed by the bowel, it occurs, not unfrequently, that they are so contracted in size as to convey the apprehension of some stricture of the canal; but the effect appears to be really owing to the unhealthy state of secretions, and to muscular contractions of the bowel irregularly occuring from irritation. It sometimes happens that air is formed so abundantly in the intestines, either from undigested aliment, or possibly from actual secretion, or both, that its effects in producing painful distension, alone serve to cause a most distressing state of complaint. The discharges are commonly not formed, but are remarkably tenacious. They assume the appearance of pitchy blackness, or are muddy, or resemble dirty clay; and when the complaint has been of long continuance, an excessive quantity of mucus is secreted in the intestines, which incorporates itself with the fæces, and sometimes exhibits almost the appearance of purulent discharge.* This mucus is to be distinguished from the gelatin-like appearance, which is seen occasionally in the evacuations, when the bowel suffers excessive irritation from an acrid purgative, or when under dysenteric affection; in which case, it appears detached from the fæculent matter, apparently almost organized from its firm consistence, and is, indeed, altogether different from the slimy accumulation before mentioned.

* "If water be poured on these fæces, the mucus separates into small flakes."

This habitual mucous secretion has always appeared to me an indication, that the morbid condition of the alimentary canal has been of long standing. In the same manner we see the urine loaded with mucus, when the bladder is diseased, or under permanent irritation from a morbid condition of its contents." 83.

We have not room for any farther notice of this Section in our author's work, but recommend it to the careful study of our brethren.

It is well known that Dr. Scudamore has directed his attention much to the function of the kidnies, as constituting a very important link in the pathology of the digestive organs. When gout is in question, the patient frequently experiences a deficient secretion of urine a short time before the occurrence of the paroxysm, the fluid itself being of a deeper colour than natural. In people of a very nervous temperament, however, the urine is sometimes passed copiously, and of a pale colour. In the paroxysm, Dr. S. has found the morbid urine much increased in specific gravity, as ranging from 1.010 to 1.015, and sometimes even to 1.035, possessing the natural acid character, invariably, when in the recent state; but, from its high degree of animalization, soon becoming putrid and alkaline. The pink dust and mucous sediments in urine, are inseparable companions of gout:

"The appearance of these sediments is entirely dependant on a faulty state of the digestive organs; and upon unhealthy assimilation." 98.

Among the *exciting* causes of gout, Dr. S. enumerates the heating and injurious qualities of *Champaign*.

"If it has power to excite a first fit, we need not wonder that it is a fruitful occasional cause, in producing the returns of the disease."

"*Cold*, with or without wet, applied to the body generally, or to the lower extremities only, especially when in concurrence with fatigue, proves, in an equal degree, exciting to the gout, in an individual who is predisposed to the disease, as to the phlegmasiæ in general; and it is by far the most frequent of the exciting causes. Cold, whether applied locally or generally, acts most powerfully when conjoined with wet; but certainly the *East wind*, by itself, is a severe and active agent." 108.

Under the head—"Proximate Cause," Dr. S. has discussed, and, as is usual on such occasions, subverted all preceding theories, in order to make way for his own. This, however, he has modestly brought forward under the title of "Ratio Symptomatum, or the theory of the symptoms, including the chemical history of the urinous sediments." In the following extract, the prominent features of our author's theory of gout will be seen.

"In a first fit of gout, the plethoric state of vessels, either absolute or relative (of which Dr. Parry has taken ample notice), appears the predominant, and often the only circumstance which can be detected in the errors of the constitution. In the returns of the disease, more or less of irregularity in the functions of the abdominal viscera becomes conspicuous; and it gradually assumes a more complicated character. In a general statement of the fact, it may be said, that the plethora which exists is of a partial kind. That determination of blood to the extremities, which, in its peculiar actions, exhibits the phenomena of gout, becomes more and more obviously connected with congestion in the whole system of the vena portarum, with a vitiated secretion of bile, costive bowels, and unequal function in the kidnies.

"The stomach is truly the medium through which the gout is created. Excess of ingesta, beyond the powers of healthy assimilation, and the supply of blood demanded for the useful purposes of the body, is the material foundation of the disease. In those instances of sudden and unexpected attack, when the patient considers himself in the most vigorous state of his health, he is pursuing free habits of living, and carelessly producing a state of repletion, which insidiously grows into a fit of gout. The increased specific gravity of the urine depending upon an increase of its solid principles, which constantly takes place in a paroxysm, appears to me one certain evidence that the blood-vessels are surcharged with blood, unhealthy in quantity, and probably also in quality. In addition to the excess of the saline ingredients of urine, so constantly found in the paroxysm, with relation to the time of health, the fact, of which I have obtained abundant proof, that *urea* is also excreted in preternatural quantity, deserves particular attention.

"A copious appearance of the pink or lateritious sediment, which is to be taken in connexion with an increased excretion of other animal principles, is an indication that the kidnies are secreting from the blood much unassimilated matter; and according to the degree and duration of this symptom, we are enabled to form a strong conclusion as to the magnitude and importance of abdominal visceral complaint. I consider that we are to view this preternatural secretion of the kidnies at once as the *sign* of disease, and as a salutary process which Nature is performing, to relieve an overloaded and faulty state of the circulation of the liver, and the organs associated in its functions." 139.

Dr. Scudamore supports his *hypothesis* (for he cannot disguise theory, after all, under the veil of "ratio symptomatum") with considerable ingenuity, and winds up thus:—

"We are now brought to the general conclusion, that gout is a disease depending upon a redundancy of blood, with relation to the powers of the circulation, particularly affecting the system of the vena portarum, and the consequent functions of the liver; together with the production of a morbid change in the secreted products of the alimentary canal in general, and of the kidnies in particular." 141-42.

Although we suspect that this theory leaves us but little wiser than before; yet, as it tends towards a rational mode of treatment, and appears to grow out of the facts and actual phenomena of the disease, at least as naturally as most of its predecessors, we shall not enter into any captious examination of its defects or weak points, in this place, though we shall make a few observations on its comparative merits presently.

We shall pass over the Sections on Diagnosis and Prognosis, in order to concentrate the more our attention on the final object of all our investigations—the *Treatment.*

The prohibition against therapeutical interference in the paroxysm of gout, by such a man as Sydenham, who was himself a martyr to the disease, and who studied all its phenomena with the most profound attention, has long and deservedly, we think, exerted a powerful influence on medical practice. "It is obvious (says Dr. Scudamore) that he derived *all* his opinions from the doctrines of the humoral pathology." This is not quite so obvious to us. We do not see any reason why the clear and comprehensive mind of Sydenham should not found some of its opinions upon the observation of facts, as well as the mind of Dr. Scudamore. Dr. S. brings forward the following passage, apparently to expose the theoretical errors of Sydenham.

"In this disease, Nature seems to have the prerogative to expel the peccant matter, according to its own method, and throw it off from the joints, there to be carried off by insensible perspiration." 173.

Now, if the reader will please to revert back to the passages just extracted from Dr. Scudamore himself, he will find a mere amplification of the above doctrine of Sydenham, not exactly totidem verbis, but certainly conveying a similar import. Dr. Scudamore, indeed, has somewhat altered the route of the *peccant* matter, and speculated on its chemical nature; but still it is unhealthy, "unassimilated matter;" and the "blood-vessels are surcharged with blood, unhealthy in quantity, and probably also in quality," with "*excess* of saline ingredients in the urine;" and to crown the whole, Dr. S. is forced to acknowledge that this "increased excretion of other animal principles" is not only a *sign* of disease, but "a *salutary* process which Nature is performing to relieve an *overloaded and faulty state*

of the circulation of the liver, and the organs associated in its functions." What, in the name of common sense, is all this but a new version of the old and ridiculed humoral pathology of Sydenham and his predecessors? Let us not then be teazed with the declamations of the moderns against the humoral doctrines of the ancients, while the former can only coin new phrases for the ideas of the latter.

Dr. Scudamore, in his commentary on the above passage of Sydenham, while he acknowledges "that nature is seeking a remedy for herself in a fit of the gout," and that her powers are generally sufficient in slight and primary attacks, yet draws a frightful picture of the ultimate effects of the disease when left to itself. But we may just hint here, that one reason why we see fewer cripples from gout now-a-days, when the work is taken from the hands of Nature, and the medicina perturbatrix is in full play, may be the erasure, from the list, by sudden deaths, apoplexies, cardiac aneurisms, &c. of a considerable number annually, *before* the sequelæ, delineated by our author and others, could become apparent. But while we deprecate strong measures of cure during the paroxysm of gout, we advocate the most energetic means of preventing the necessity for this painful, though salutary effort of Nature. In the following passage, there is a saving clause following the word "unless," which enables Dr. Scudamore to accommodate all parties.

"I would assume it, therefore, as a principle, that we should attempt the prevention of a fit of gout, if warned of its approach; and interrupt its progress when formed, *unless* such a state of the constitution exist, that the gout has taken the place of another more serious disease; or may be expected to prevent one which is threatening, and more to be dreaded than itself; but even in this case, it is incumbent upon us to moderate the violence of symptoms; to study and fulfil particular indications; and carefully to estimate the balance of the present evil with the expected good." 175.

Now the question is whether we may not, by interrupting the progress of the fit when formed, always endanger the supervention of some other form of the complaint, or its conversion into some other disease. This we firmly believe; and the sanction of a Parry, who studied in the very head quarters of gout, and drew his conclusions from a wider field of experience than any author now living, is a sufficient shield for our creed. But we shall now hasten to lay before our readers a comprehensive analysis of Dr. Scudamore's Therapeutics.

I. *Treatment of the Premonitory Symptoms.* Dr. S. thinks that the threatened attack may be averted, or if not, the subsequent paroxysm rendered milder, by the following means: viz. *general bleeding,* if the true inflammatory diathesis be present; or if congestion of the vessels of the head, liver, or other internal organ be indicated, without corresponding increase in the pulse, then *local* detractions of blood. We believe that no rational practitioner, of the present day, would withhold these means, under the same indications, whether there was or was not gout in the system.

When there is a tendency towards hæmorrhoidal discharge, it should be promoted, by aloetic and saline purgatives. The costiveness, so usual a forerunner of gout, should be removed by active purgation with calomel, antimony, and ex. colocynth.

"If a furred state of tongue, with heart-burn, nausea alternating with a craving appetite, and acid eructations be present, an emetic of ipecacuan should be administered." P. 177.

If the threatening symptoms continue, the means recommended hereafter for the paroxysm itself should be put in force; and if, after the employment of suitable evacuations, the internal secretions remain in a vitiated state as indicated by the fæces and urine, "small unirritating doses of mercury at proper intervals," with a gentle aperient once a-day occasionally conjoined with a stomachic bitter, will be found useful. To these means must be added a regulated diet, or even abstinence; exercise; repose of mind; early hours; in short, a change of the lædentia for the juvantia.

II. *Treatment of the Paroxysm.* Blood-letting is not so allowable in this as in other phlegmasiæ; since—

"It usually happens in gout that the increased excitement affects the *nervous* system much more than the heart and arteries; and, as I have before stated, the redundancy of blood appears to belong rather to the circulation of the vena portarum, than to that of the general system. It may also be assumed as a practical fact, that this kind of partial plethora is more favourably and effectually removed by purgatives and diuretic medicine than by the detraction of blood itself." P. 179.

General blood-letting, indeed, Dr. S. considers wholly unnecessary, and quite ineffectual in the removal of the *local* inflammatory action. Nevertheless, when the *general excitement* is strongly marked, *general bleeding* will be indicated—and *a fortiori,* if an internal organ be inflamed, for then the gout must be totally disregarded. It is obvious that the above precepts are entirely applicable to all the phlegmasiæ, and bear little, after all, on the particular treatment, if indeed there be any *particular* treatment required in gout. Local congestions or inflammations in gout, as in all other cases, are to be met by local abstractions of blood.

III. *Emetics.* From our author's own experience, he does not advise the employment of emetics—"*unless an evacuation of the stomach in a full degree is obviously required, from its being pointed out by indications* of irritating contents." 182. This is one of those sound but very common truths, to which no man can possibly refuse his assent.

IV. *Cathartics and Diuretics.* On these Dr. S. chiefly relies for the successful treatment of the paroxysm.

"Occasional doses of calomel, in conjunction with antimonial powder, compound extract of colocynth, and a little soap, fulfil, in the most useful manner, the first part of the intention (purgation) which I have expressed; and they should be repeated each night, or each other night, according to the degree of vitiation which the bilious and other matters from the bowels appear to possess; and according to the advantage derived." 185.

In co-operation with the above, our author has experienced "the most remarkable success from a draught composed of magnes. gr. xv. ad xx.; magnes. sulph. ʒi. ad ʒii.; aceti colchici ʒi. ad ʒii; with any distilled water the most agreeable, sweetened with any pleasant syrup, or with xv. or xx. grs. of ex. glycyrrhizæ." When much feverish heat of skin has prevailed, he adds twenty or thirty grains of potass. carb. accurately neutralized with lemon acid. In this latter case he prefers the carbonate of magnesia to the calcined, using a larger proportion. This draught should be repeated at intervals of four, six, or eight hours, according to the freedom of its operation, and the urgency of the symptoms; the purgative and diuretic medicines being actively administered, "until the gouty inflammation subsides, and so long as the urine, which is first passed in the morning, retains a high specific gravity, or deposits sediment." 187.

V. *Mercurials* are only given as purgatives and alteratives, as stated in the preceeding section, but never to excite mercurial action in the system.

VI. *Pretended Specifics.* It is sufficient to say that our author very properly and very ably exposes and condemns the whole list of pretended specifics in gout, from the *Eau Medicinale* of Husson down to the *vinum colchici* of Home.

"With the effects of elaterium and opium, I am the least acquainted; but I have had abundant opportunity to know that each of the other medicines, sooner or later, disappoints the patient of his expected cure, rendering merely a palliative assistance, and keeping the disease dormant for a time only, so that it is left to prey on the constitution with more lasting and serious ill effects." Page 198.

The following observation of Dr. Sutton, on this remedy, is worthy of attention.

"In the use of this medicine, also, it must be observed, that the benefit is not connected with a small dose of opium; but the quantity is defined by its producing a complete cessation of pain." *Tracts.*

"I have myself found the use of this medicine remarkably successful in its crude state, and when joined with a small dose of antimonial (in preference *James's*) powder. The patient being furnished with twelve pills, each containing one grain of *crude* opium and half a grain of James's powder, may be desired to take one, two, or, if pain be very severe, even three at bed-time, as the first dose, and repeat one every hour or two afterwards, according to the degree of pain; this being the only regulation as to the quantity to be employed, when no contra-indications are present." 224.

Dr. Scudamore informs us that he has had many opportunities of ascertaining that the Lancastrian, or *black-drop,* agrees with many individuals much better than the common preparation of opium, disturbing less the stomach, during its immediate operation, and the head on the following day. Our author also speaks in favourable terms of Mr. Battley's liquor opii sedativus, as producing less inconvenience to the nervous system than other preparations of opium. He recommends it in doses similar to those of the tinct. opii of the Pharmacopeia.

"The pulvis ipecacuanhæ comp. is also an excellent form of opiate; and when joined with the use of saline medicine, sometimes proves more useful than any other preparation."

The other narcotics are inferior in virtue to opium; but may be occasionally useful as adjuvants, where idiosyncrasy forbids the latter. Stramonium in such cases is the best succedaneum that our author has found for opium.

On the dietetic and moral management of the patient, during the paroxysm, Dr. Scudamore's remarks are judicious, but in unison with general experience. We shall therefore pass on to an important topic.

*Local Treatment in the Paroxysm.* It is on this point, we believe, that modern improvement is conspicuous; chiefly by steering a middle course between the flannel of Cullen, and the cold of Kinglake.

> " The inflammation of gout, (says our author,) has *never* been treated upon *fixed* and *regular principles.* It has most commonly been left to its own injurious course, unchecked and unrelieved." 241.

Upon this we would remark, that each author considered his principles as *fixed* and *regular* as Dr. Scudamore's; and it would be too much to expect, that the latter system alone shall stand, like a rock of adamant, and defy the waves of Time.

*Leeches.* Local bleeding, however performed in gout, Dr. S. looks upon as not only unnecessary, but "in most instances injurious." We consider this to be by no means authorized by the evidence of facts. A host of authors have not only sanctioned but recommended the measure; and notwithstanding the constitutional nature of gout, we believe that the violence of its local manifestation may often be safely and efficaciously moderated by leeches, especially in primary attacks, and where neither the constitution is broken down, nor the texture of the parts much deranged by repeated paroxysms.

Dr. James Clark, of Rome, on his way to this country a few months ago, directed his attention, at the Val de Grace Hospital in Paris, where M. Broussais presides, to this subject; and his notes, which we have had the advantage of perusing, bear ample testimony to the beneficial effects of local bleeding by leeches in the inflammation of gout. Indeed, the distinguished physician above-mentioned, uses scarcely any other *local* treatment in this complaint, either in the vast establishment over which he presides, or in private practice.

> " It has always appeared to me, (says Dr. Scudamore) to be a correct and sound principle of practice in *local inflammations of every kind,* that, whenever their violence is such as to influence the action of the heart and arteries in any considerable degree, the abstraction of blood should be made from the arm, *rather than from the part affected;*' but, that when the inflammatory action is almost entirely local, the depletion of the vessels should be local also." 242.

We look upon the above principle of practice to be very *imperfect,* to say the least, and, indeed, to speak candidly, we consider it *unsound.*

The general excitement, in this case, grows confessedly out of the local, as its *cause*; and yet, Dr. Scudamore's principle would direct us rather to obviating the *effect*, than to removing the cause on which it depends. When local inflammation has kindled up general fever, we are by no means to direct our attention exclusively, or even in preference, to the *latter*. Our local depletion should *then* be equally active, or indeed more so than before; the general depletion being put in force, merely as an auxiliary to counteract the constitutional effects, while topical depletion is to be vigorously pursued to remove the cause of all. We appeal to every man of experience and observation, for a judgment on this point of discrepancy between our author and ourselves.

Of vesicatories and irritants in acute gout, Dr. S. cannot speak from experience; and, indeed, they require no notice. We were somewhat surprised to find a page of Dr. Scudamore's Practical Treatise taken up with Lucian's Tragopodagra, copied from Guilbert, and which has been wisely omitted in Dr. Johnson's last translation of that able work.

*Warmth* is condemned by Dr. Scudamore, as belonging to the "worst part of the ancient practice." And yet his own "tepid evaporating lotion," might, without much difficulty, be proved to be a branch from the same stem. *Hot* bathing of the extremities in acute inflammation of gout, no one would surely advice.

"When the cleanliness and softening of the skin of the surrounding parts, together with that immediately affected, are desired during the paroxysm, the use of free sponging with *tepid water* is much to be preferred." 247.

*Poultices.* Although our author considers that a free employment of relaxing poultices, "made in the ordinary way," has the disadvantage of increasing œdematous swelling, and subsequent debility; yet, "he has found great cause of satisfaction in the occasional use of a poultice, made with bread which has been scalded with boiling water, pressed through a strainer to dryness, and again rendered of sufficiently soft consistence by means of the lotion which shall presently be described. It is then to be applied, thick in quantity, very moist, just tepid, and without any intermediate covering between it and the part affected." When the hands or feet only, are the seat of complaint, Dr. S. uses these poultices only at night.

"But for the relief of the knee and elbow, I have found the poultice to be very much more useful than the lotion applied by compresses; and have directed it to be repeated twice or thrice in the twenty four hours, according to circumstances." 250.

For a short time after the discontinuance of the poultice, the part should be covered with a single layer of flannel.

Dr. S. has given the form of Pradier's cataplasm, as translated by Dr. Johnson from Guilbert's work, and thinks that, with the omission of some of the ingredients, it deserves trial in obstinate cases of chronic gout.

*Other Modes of Evaporation.* Dr. Kinglake's revival of cold in gouty inflammation is wisely condemned by our author, who observes, that "from all he can learn of the practice of applying cold water, the relief is never so certain as the danger" 252. The following is Dr. Scudamore's favourite topical application in gout; the local treatment being, however, in his opinion, of secondary importance, though still a point of great magnitude.

"I have now the satisfaction to state, that in about one hundred and thirty cases, I have made very free use, and with the best success, of a lotion composed of one part of alcohol, and three parts of mistura camphoræ; applying it to the affected part by means of linen rags, first rendered just agreeably lukewarm by the addition of a sufficient quantity of boiling, or very hot water. In this manner, a prompt convenient method is afforded of using the lotion, on the principles on which I recommend its adoption. The rapidity with which the alcohol alone would evaporate, is advantageously retrained by the dilution with the camphor mixture; and the warming it, by the addition of hot water, preserves it from that escape of the volatile parts, which the sudden heat of the fire would occasion. In using the lotion, if it be applied either hot or cold, the intention of the remedy is considerably frustrated; and I have observed, that from being made too warm, its operation has been injurious, rather than beneficial. If the temperature be measured by the thermometer, I may state that it ought not to be less than 75 degrees, nor more than 85 degrees. I consider, however, that the expression of *just agreeably lukewarm*, is a secure and sufficient direction to the patient. The linen compress, constantly kept wetted with the lotion, should consist of six or eight distinct folds, one laid upon another; and the slightest and coolest covering only should be used in addition. The effects of this lotion, when it has been attentively employed, have been most satisfactory, and have really answered my warmest expectations." 253.

Dr. S. asserts, that he has not, in any one instance, seen the least tendency in this lotion to produce retrocession of the arthritic inflammation, and that in only three solitary cases had it been "laid aside from disapprobation." The odour, of the lotion is pleasant and refreshing; but the linen compresses should never be allowed to become dry; one set being alternated with another, when the part is much heated, for the advantage of a cooler medium of application. When the gouty inflammation is nearly overcome, or when it happens that the lotion, applied by compresses, ceases to produce comfortable sensations, and, on the contrary, occasions chilliness or numbness, the method is to be changed, and "the parts should be sponged with the lotion frequently, and left damp, with or without subsequent covering of a light kind, as circumstances may direct." 256.

The good effects of this evaporating lotion, however, are chiefly discoverable when the inflammation is seated in the more superficial tissues and structures; in proportion as the disease is remote from the surface, its operation will prove less efficacious, and in opium (the proper indications being fufilled) our superior confidence must be placed. In these latter cases, our author employs, in preference to the lotion, a cataplasm made with the lotion, directing it to be renewed thrice in the twenty-four hours.

While we can corroborate, from personal observation, most of Dr. Scudamore's commendations of this evaporating lotion, it is impossible not to see that our author overrates its importance, as a favourite primogeniture of his own. However original the *form* of the lotion may be, the same cannot be said of the principle. Both Boerhaave and Cadet, and, for aught we know, many others, have witnessed the powerful influence of "aspersions of tepid water on the gouty limb,"* the operation of which, it is evident, was precisely what takes place in Dr. Scudamore's mode of application. We do not make this remark in derogation of our author's remedy, but to shew that it is exceedingly difficult, in so obstinate a disease as gout, to bring forward any curative measure which has not been tried, in some shape or other, by our predecessors. On the contrary, we consider Dr. Scudamore's lotion as the best formula that has ever yet been proposed to the profession.

Our author makes several judicious observations on the period of convalescence from an arthritic attack. In

* See Guilbert's Work, page 148 and 151; or, Dr. Johnson's Translation, page 76.

the great majority of cases under his own managemen , bitters, and tonic medicines, of every description, have been unnecessary; on the other hand, he has preferred a mild course of aperients and alteratives, in conjunction with a regulated diet and general regimen. Where simple debility, however, succeeded a long, and exhausting effort of Nature, or an improper interference of Art, he has used, with advantage, the following combination:

℞. Calumbæ radicis concisi ʒj ad ißs. Cascarillæ corticis contusi ʒij ad ℥ß. Rhei radicis contusi ℈j ad ℈ij. Cardam. sem. contrit. ʒß ad ʒj. Aquæ ferventis octarium dimidium. Macera per horas duas, et cola.

℞. Hujus infusionis ʒx ad ʒxv. Tinct. aurantii ʒj. Sodæ carbonat. gr. x ad xv. Misce fiat haustus bis quotidie sumendus.

The ammoniated tincture of iron, where no tendency towards inflammatory plethora exists, may be usefully employed. Another form of tonic stomachic medicine, which our author has found to agree well with gouty and dyspeptic stomachs is, "the union of decoct. aloes, compos. infus. gent. compos. et mist. camphoræ, with a moderate addition of spir. ammon. compos. to be given once, twice, or thrice in the day.

As an alterative pill, to be given with the view of exciting healthy secretion, Dr. S. recommends five grains, every second night, of the pil. hydrarg. sub. compos. as preferable to the pil. hydrargyri. For the local sequelæ of gout occasioning lameness, with œdema, coldness of skin, inability to bear the weight of the body, with a shrunk condition of the muscular fibre, Dr. S. recommends, in addition to the morning sponging and subsequent friction, the application of the following liniment:

Tinct. lyttæ ℥ß liniment. camp. comp. liniment. sapon. compos. āā ℥iß. M.

The foregoing didactic precepts relative to acute gout, are illustrated by a series of cases occupying sixty pages of closely printed letter press; and this is one of the items by which our author has, we think, very injudiciously swelled the work to a size and shape so unwieldy, as to cripple its circulation completely.

*Chronic Gout.* This form of the disease is characterized by our author in the following definition:

"Inflammation and pain more slight, irregular and wandering, than in the acute; faint redness of surface; much permanent distension of parts, or continued œdema, and impaired moving power, without critical indications of its terminating; commonly associated with a morbid state of the digestive organs, a languid or oppressed circulation, and much nervous irritation in the system." 14.

Although chronic is most commonly an ultimate consequence of acute gout, yet it sometimes occurs in subjects who have never experienced the acute form. A great variety of anomalous constitutional indispositions attend it, particularly of the dyspeptic class, with fugitive cramps and spasms in various parts of the body, which are very distressing to the patient.

"An exceeding irritability marks the temper. The mind is hypochondriacal; imaginary evils disturb the judgment, and shake the resolution on trifling occasions. Palpitations affect the heart; and the sensations, described as flutterings, are still more frequent. Either from pain or uneasiness, the sleep is disturbed and unrefreshing. I have met with female gouty patients in particular, who are so exquisitely sensible to the vicissitudes of atmosphere, that instantly on the change of the wind to a cold quarter, and especially if accompanied with moisture, they feel wandering pains in the limbs; and indeed are so susceptible, that their nerves are true barometers." 334.

At page 343, Dr. Scudamore hazards an opinion which, we think, is by no means supported by any pathological principles or facts yet ascertained; namely, that gouty and rheumatic inflammation can never exist in the same part, and at the same time. He admits that, "certainly we find gout and rheumatism occasionally existing in different parts of the body at the same time." Now, on what principle can he deny their combination in the same part, when he allows their simultaneous existence in the same body, seeing that he does not consider them as both constitutional diseases, rheumatism being, in his opinion, a local disease? The only plea of incompatibility of cotemporaneous action in the two diseases is thus taken away by our author himself; and granting that the two inflammations were distinctly specific, and both constitutional in their origin, we are very far from placing implicit credence in the celebrated Hunterian dogma relative to the incompatibility of their simultaneous existence. As for any precise diagnostic marks, by which gout can be distinguished from rheumatism, when they are both in action at the same time, and in the same person, we appeal to clinical observation for their fallacy; at least, we acknowledge our own complete incapacity for such a task, and we think we have seen some cases of what is commonly, though perhaps erroneously, termed " rheumatic gout," which would have puzzled Dr. Scudamore himself to draw the line of demarcation between the contending diseases.

" Nevertheless (says Guilbert) we every day meet with examples of gout and of rheumatism, resulting from a *combination* of those causes; and so, in truth, do we see rheumatic gout, as well as gouty rheumatism, mixed affections, whose characters partake of both diseases, and whose treatment requires correspondent modification."

*Treatment of Chronic Gout.* In acute diseases, as Dr. Scudamore well observes, the bold hand of the Empiric, or some happy effort of Nature, may sometimes be speedily successful; but when the chronic form of disease is deeply established in the system, no pretended universal, or even expeditious method of cure, can have any just claim to our regard. Our author, in this place, draws our attention to three modifications of chronic gout, which he thinks are necessary to be discriminated from each other, for the sake of a more successful treatment. The principal features of these modifications, with the appropriate methodus medendi, as laid down by our author, we shall endeavour to present to the reader.

I. In the nervous temperament, where the arthritic diathesis may be strong, yet the powers of the system unequal to the production of regular gout, the internal functions will be found weak and irregular, with much morbid nervous sympathy. Here, although a comparatively too full diet may produce slight inflammatory actions, with pain, swelling, and difficult motion of parts, yet general bleeding will seldom, our author thinks, be necessary.

" The employment of a purgative diuretic medicine in the saline combination, such as before recommended, with the occasional interposition of a dose of hydr. submur. et pulv. jacobi, and the abstraction of all heating stimuli, will usually constitute a treatment of sufficient activity." 346.

Pain and nervous irritation must be soothed by gentle anodynes at bed-time; and, in such cases, Dr. S. recommends stramonium and lactucarium, or the latter in combination with small doses of the pulv. ipecac. compos.; these, he thinks, are preferable to opium and hyoscyamus.

" The local treatment is to be conducted exactly on the principles formerly described." 346.

Inflammatory tenderness being removed, friction and bandages will be eminently useful. In respect to the more permanent measures, the internal functions, more especially those of the liver, kidnies, and digestive organs, must be watched carefully, and steadily improved, both by regimen and remedies. In the summer season, the use of the warm sea bath twice or thrice a week, will form a valuable auxiliary.

II. "The consequence of acute gout, when its repeated invasions have impaired the energy of the constitution; and from the weakened circulation which is induced, the chronic diseased action alone takes place. The functions of the internal viscera are more or less deranged; and the nervous system is much disturbed.

"In this example, we commonly see that the primary character of constitution remains to so great a degree, that signs of plethora are often manifested; and slight local inflammation is readily aggravated by the injudicious use of stimulants. Under these circumstances of vascular susceptibility, in conjunction with languid powers, the treatment which has been stated in the preceding example is generally applicable. The alterative aperient plan will, however, sometimes be required to a greater extent, and for a longer continuance. The state of the secretions will be the true guide to the practice which should be adopted." 348.

In certain states of debility and general disorder into which some gouty invalids decline, or in anomalous cases of disease, where gout is suspected in the system, without having made any *local* demonstration, it has been usual to invite a fit of the gout "by various modes of stimulating treatment." This our author thinks a hazardous practice, and so do we; but, in such cases, we certainly should be for encouraging the local disease by mild means, while we still agree with our author, that the essential treatment must "consist in a regular and persevering attention to the chylopoietic functions, both by means of medicine and regimen."

In respect to Bath waters, Dr. Parry informed our author, that in no form whatever were they beneficial during the paroxysm, or in any inflammatory disposition in the intervals. In that dyspepsia, however, which is joined with a languid circulation and want of nervous energy, but without any inflammatory tendency in the organic system, "these waters appear calculated to be eminently useful."

"In most instances, and indeed almost without exception, the waters of Cheltenham prove highly beneficial to gouty persons; and particularly when joined with a medicinal alterative and regulated regimen. The water, No. 4, is that which is most suited to the gouty patient; and especially in the first instance."—"The waters of Leamington are entitled to similar praise with those of Cheltenham, but their aperient effect is less active."

III. This is the chronic state of gout arising out of repeated acute attacks, with local changes of structure, irritable nervous system, but healthy condition of the natural functions.

"With this form of chronic weakness, rheumatism is often blended, and the patient is extremely susceptible to every vicissitude of weather, and especially to wet, and damp cold air."

"In the pains and frequent threatenings of inflammation, which, under these circumstances, continually occur, the combination of a narcotic and a sudorific appears particularly useful; and for this purpose, the compound powder of ipecacuanha, in small doses, twice or thrice in the twenty-four hours, often proves a valuable medicine, strict attention being at the same time paid to the proper action of the bowels and kidnies. In this form of the complaint, however, I must not lose sight of the recommendation which is due to the combination of stramonium and lactucarium, from which I have repeatedly seen the best effects derived." 362.

It is in these cases that our author has seen much benefit result from a system of tepid bathing, especially in the Buxton waters, with friction.

This section of our author's work is illustrated by between fifty and sixty pages of cases, which we shall, of course, entirely pass over. We cannot but lament this supererogation. We conceive that one-fourth of the space would have been quite sufficient for illustrations of this kind, after the minute detail of principles and indications previously laid down.

We now come to the last division of the subject; namely,

*Retrocedent Gout.* This, our author thinks, is of rare occurrence, excepting from mismanagement or want of care. The transference is most apt to attack the stomach or intestines, or both in succession. In the *former* it produces exquisite pain, spasm, and sickness; in the intestines, enteritis, in its worst form, too frequently results. In either case the danger is pressing; and if relief be not obtained, death soon closes the scene. If the translation be to the brain, in its worst form, apoplexy is produced, and is generally fatal.

"Sometimes (says Dr. Cullen) the internal part is the heart, which gives occasion to syncope; sometimes it is the lungs, which are affected with asthma."

Dr. Scudamore has never seen any instances of translation to these latter organs. But others have seen numerous examples of such.* It is not, however, in sudden translations, during a paroxysm of gout, that we are to look for the lesions of internal organs from that source, but to the slow conversions of external gout into chronic inflammations and irritations of internal structures. This wide and important field Dr. Scudamore has left almost without cultivation, while the volume has been swelled beyond all bounds, by minute, and, in many instances, unnecessary details of common and open forms of the disease.

*Causes of Retrocedent Gout.* The most frequent are vicissitudes of temperature applied to the body generally, or cold to the affected parts. Here Dr. Scudamore relates several interesting instances illustrative of this cause.

"When cold is the hurtful agent, the internal symptoms which are produced are probably, for the most part, of an inflammatory nature. I have formed this opinion from such cases as have come under my own observation; and from the general information which I have collected." 434.

Gout is often rendered retrocedent by the agency of certain stimuli suspending the external gouty action, as, for instance, in the use of *Eau Medicinale,* &c. Indigestible food, violent passions of the mind, and other accidents, have also considerable power in interrupting the regular process of gout, and transferring it to a predisposed internal organ or tissue.

*Diagnosis.* To distinguish between spasm and inflammation, in retroceded gout, is often a task of no small difficulty.

"In an attack purely spasmodic, the rigidly contracted state of the abdominal muscles, and the relief which is afforded by strong pressure, are very distinctive. When it is purely inflammatory, the tender state of parts to the slightest weight or pressure; the more regular diffusion, yet greater fixedness of the pain; the sympathetic fever which is instantly produced; and, indeed, the very physiognomy of the patient in the comparative situations of attack, will, to the experienced practitioner, be a description of the nature of the disease. The state of the pulse, as whether small and indistinct, or full and oppressed, or in vigorous action in any way, will materially direct the judgment; and the state of the skin and features, whether cold and collapsed, or in contrary states, is a guidance of importance. Dr. Cullen, and authors in general, appear to have considered it as a settled axiom of practical doc-

---

* See a remarkable case related by Guilbert, page 28 of Dr. Johnson's Translation.

trine, that debility and spasm, and not inflammatory action, seize the internal organ in the case of retrocedent gout.

"A perfect conviction prevails in my mind, that, in a genuine example of retrocedent gout to an internal organ, inflammatory action is the more common occurrence, and that spasm alone is comparatively rare. The mixed action of spasm and inflammation may however, be expected to happen still the most." 436.

*Treatment*. In every case of retroceded gout Dr. Cullen directed a stimulating treatment, and this is still the popular custom in this country. Every gouty invalid has his bottle of Cogniac, Madeira, or Usquebaugh, safely deposited for immediate use, when the enemy attacks the stomach. When the retrocession consists purely of spasm, it is all very well. But when inflammation exists, the practice is extremely questionable, if not entirely objectionable. The life of our patient hangs on the discrimination which we exert.

"If retrocession have been excited by indigestible food, the sickness which is present, and the appearance of the rejected matter, point out that the vomiting should be promoted. An emetic of ipecacuanha is well adapted for this purpose; and its operation is to be assisted by draughts of warm water in the usual manner. If the pain be thus relieved, the bowels should next be acted upon; and five or ten grains of calomel should be given as an immediate dose. As soon as the stomach can retain a purgative medicine, the draught, p. 186, may be given every three hours, until a full operation is produced; or the combination of sulphate of magnesia and infusion of senna, with an aromatic tincture, may by some be preferred. Whatever medicine of this description may be chosen, the dose should be repeated at short intervals, until a full effect is produced. The aid of an injection will occasionally be required. If, however, violent pain should still continue, after the stomach has been cleared of its contents, tincture of opium, in a dose from forty to one hundred drops, may be given without hesitation; it must be repeated also in free doses, at an interval of half an hour, or an hour, until pain and spasm cease, or satisfactorily abate; and at the same time, purgatives, which will have their effect delayed, but not prevented by the opiate, must, on no account, be omitted. Pills of calomel, colocynth, and soap, constitute the form of active remedy which is most to be recommended; and the fluid purgative should follow their administration." 438-39.

When exposure to cold, or violent stimuli have been the exciting causes, in a patient whose circulation is strong, and habit plethoric, we may dread inflammation, and our author boldly recommends the abstraction of sixteen, twenty, or thirty ounces of blood from the arm, to be repeated according to circumstances. When the retrocession has been on the bowels, we must treat it as enteritis, and that with promptness and decision, always endeavouring to solicit back the gouty inflammation to the extremity which it last deserted.*

In gouty apoplexy from retrocedent gout, "copious bleeding, to the extent that the pulse permits, is the only remedy that can save the patient." Cold evaporating lotions should be freely applied to the head, and stimulating applications, with heat, to the lower extremities.

A number of very interesting cases follow this section, in illustration of the diagnosis between the spasmodic and inflammatory forms of retroceded gout, and also of the treatment in both. These we must leave untouched, as our object has been rather to lay down principles, than details or narratives.†

The highest compliment we can possibly pay to a work, is a full analysis of its contents, sparingly interspersed with either censure or applause. In the first place, it is a proof that we have read the work attentively—which is more than every Reviewer can say! *Secondly,* the appropriation of a large space to one publication, when so many are daily issuing from the press, shews our comparative estimation of its value. *Thirdly,* it is no small favour to an author, to allow him to plead his own cause at the public tribunal, almost entirely unembarrassed by critical quibbles and cross-questions; a mode of procedure which enables the reader, also, to form his own unbiassed judgment of the merits of the work.

Upon these principles, we think Dr. Scudamore has reason to be satisfied. But we will go a step farther. We will state it as our belief, that the work just reviewed, is the best book on the subject, in the English, or in any other language, ancient or modern. Dr. Scudamore has fair cause to be proud, in thus bearing off the palm from a host of able and willing competitors, in an æra of enthusiastic research, and when every avenue to medical fame is crowded with talent almost to suffocation.

But as, in human life, there is no unmixed good; so,

---

* *See* an admirable suite of practical observations on the subject of retroceded gout, in the translation of Guilbert's work, from page 38 to page 96.—The continental physicians are bolder than the English in these cases, and have more resources. We think too, that they are more judicous than ourselves, in the vigorous local depletion by leeches from the vicinity of the part invaded by retroceded or misplaced gout.

† The subjects of Gravel and of Rheumatism are reserved for Review in a subsequent Number of the Journal.

in works of progressive science, there can be no absolute perfection. To a medical author, therefore, of sound and philosophic mind, the strain of unqualified eulogy never can be grateful, since it must always indicate one of three things; the partiality of friendship, the alloy of insincerity, or the dereliction of independence. It is surely desirable that these should be avoided in the examination of scientific productions, and under this impression, we shall beg leave to make an addional remark or two, in closing this extended analysis.

We are disposed to think, that Dr. Scudamore's work is not quite free from the sins both of omission and commission. It appears to be too minute, and not sufficiently comprehensive. It is too minute in the delineation of shades and modifications of disease, depending for their existence on the infinitely varied constitutions of individuals, and ever-varing causes and circumstances of the case. In this way, distinctions may be multiplied without end; and what is worse, without object or use. The progress of a science is to be accelerated by men of talent and observation seizing the distinctive characters of disease, and delineating them with clearness and fidelity, so as to draw from them sound *principles* and *indications* of treatment. Where endless varieties are traced, in the circumlocutory narratives of *cases*, the young are frightened, and the old are tired by the detail.

But while the common and *ostensible* features of gout are pourtrayed with more than German diffuseness, we think that the *constitutional* maladies dependent on the gouty diathesis, are proportionally neglected; and hence the work is not near so comprehensive in its plan, or enlarged in its views, as that of M. Guilbert.

Should the public think with us, on these two cardinal points, (and time will soon decide the question) Dr. Scudamore will do well, in a future edition, to profit by these friendly hints;—and above all, to lessen the number of cases, simplify the descriptions, and lay down clear and well defined principles of treatment.

One word more, in explanation of so long a review of a *third* edition. In the first place, from circumstances unnecessary to be detailed, the work has never been *analytically* reviewed in any Series of this Journal. In the second place, the price and size of the publication preclude its circulation among one-fourth of the readers of this Journal, and we are unwilling that so valuable a work should not diffuse its knowledge beyond the immediate sphere of its own presence.

